# Non-Destructive Corrosion Inspection of Reinforced Concrete Using Ground-Penetrating Radar: A Review

**DOI:** 10.3390/ma14040975

**Published:** 2021-02-19

**Authors:** Ksenija Tešić, Ana Baričević, Marijana Serdar

**Affiliations:** Department of Materials, Faculty of Civil Engineering, University of Zagreb, 10000 Zagreb, Croatia; ksenija.tesic@grad.unizg.hr (K.T.); marijana.serdar@grad.unizg.hr (M.S.)

**Keywords:** ground-penetrating radar (GPR), non-destructive techniques (NDT), corrosion of reinforcement

## Abstract

Reduced maintenance costs of concrete structures can be ensured by efficient and comprehensive condition assessment. Ground-penetrating radar (GPR) has been widely used in the condition assessment of reinforced concrete structures and it provides completely non-destructive results in real-time. It is mainly used for locating reinforcement and determining concrete cover thickness. More recently, research has focused on the possibility of using GPR for reinforcement corrosion assessment. In this paper, an overview of the application of GPR in corrosion assessment of concrete is presented. A literature search and study selection methodology were used to identify the relevant studies. First, the laboratory studies are shown. After that, the studies for the application on real structures are presented. The results have shown that the laboratory studies have not fully illuminated the influence of the corrosion process on the GPR signal. Also, no clear relationship was reported between the results of the laboratory studies and the on-site inspection. Although the GPR has a long history in the condition assessment of structures, it needs more laboratory investigations to clarify the influence of the corrosion process on the GPR signal.

## 1. Introduction

Every structure, depending on its intended purpose, must be designed and constructed so that during its lifecycle it fulfils the basic requirements for structures and other requirements, namely, the conditions prescribed by the Building Act [1]. Unfortunately, experience has shown that a large number of concrete structures show significant signs of degradation after only 20 to 30 years due to the joint action of mechanical and environmental effects [2]. The causes of degradation are mainly the consequence of corrosion, which on a global scale increases the annual maintenance costs to more than 3% of the world’s Gross Domestic Product (GDP) [3]. The maintenance and strengthening of bridges in Europe alone require ₤215 million, not including the costs of redirection and organization of traffic [4]. The unsystematic approach to maintenance, especially of infrastructure, contributes to its premature deterioration and has a negative impact on safety and reliability. Particularly worrying is the fact that today, the resources invested in maintenance and repair are higher than the cost of construction [5]. The question therefore arises: how to stop or delay the degradation of global infrastructure?

The concern resulting from the problems outlined led to the development of strategies to mitigate the consequences of the corrosion process. At the design level, strategies are mainly aimed at improving the durability properties of the concrete cover in terms of its thickness and quality [6]. Other strategies aim at the preventive use of corrosion inhibitors, corrosion-resistant steel or other surface treatments [7,8,9]. However, these have limited ability to solve the corrosion problem of existing structures.

One of the most promising approaches to delay the degradation of existing structures is the extensive use of non-destructive techniques (NDT). Increased inspection frequency and coverage of larger inspection areas could lead to timely detection of deterioration and, in sum, better decisions in the maintenance of the structure. In this regard, the progress in the development of NDT methods towards visualization of results leads to the increased use of advanced NDT methods in the future [10]. Many NDT methods are currently available; however, this paper focuses on the application of ground-penetrating radar (GPR) for corrosion inspection of reinforced concrete structures.

Originally, the radar was designed for military use [11]. Today, its application has expanded to various disciplines, such as civil engineering, hydrogeology, archaeology, etc. [12,13]. When combined with other non-destructive methods, it is feasible for evaluating the condition assessment of the concrete structures [14,15] with increased effectiveness and speed of inspection. The laboratory investigations have shown that the corrosion process could be monitored based on observing the changes in the GPR signal [16,17,18,19,20]. Moreover, GPR has a history in the assessment of corrosion and corrosion-related pathologies for on-site inspection [21,22,23,24,25]. The main difference between these two approaches is that the laboratory investigation was mainly focused on discrete corrosion characterization. The on-site investigation is based on the observation of several simultaneous effects, namely the variation of moisture, chlorides, and the formation of corrosion products and cracks.

Previous studies have focused on reviewing the general application of GPR in civil engineering [26,27] or have focused on on-site inspection for a specific type of construction [28]. To date, there is no comprehensive critical study that evaluates the use of GPR for corrosion assessment of reinforced concrete. The main objective of this review paper is to identify all relevant publications on corrosion assessment of reinforced concrete using ground-penetrating radar. The authors have attempted to gain more understanding of the relationship between laboratory testing and the application of GPR on-site. This prompted the authors to organize the paper into the following sections. Section 2 presents the details of the literature search in terms of the databases used, the search terms and the rationale for the publication screening. Section 3 deals with the main topic. It is introduced with the corrosion process and the main principles of GPR, presenting the characteristics for corrosion monitoring. Furthermore, it is divided into sections dealing with laboratory and on-site inspections, with each section ending with conclusions. Finally, Section 4 summarizes the work with recommendations for future studies.

## 2. Methodology

**Figure 1:**
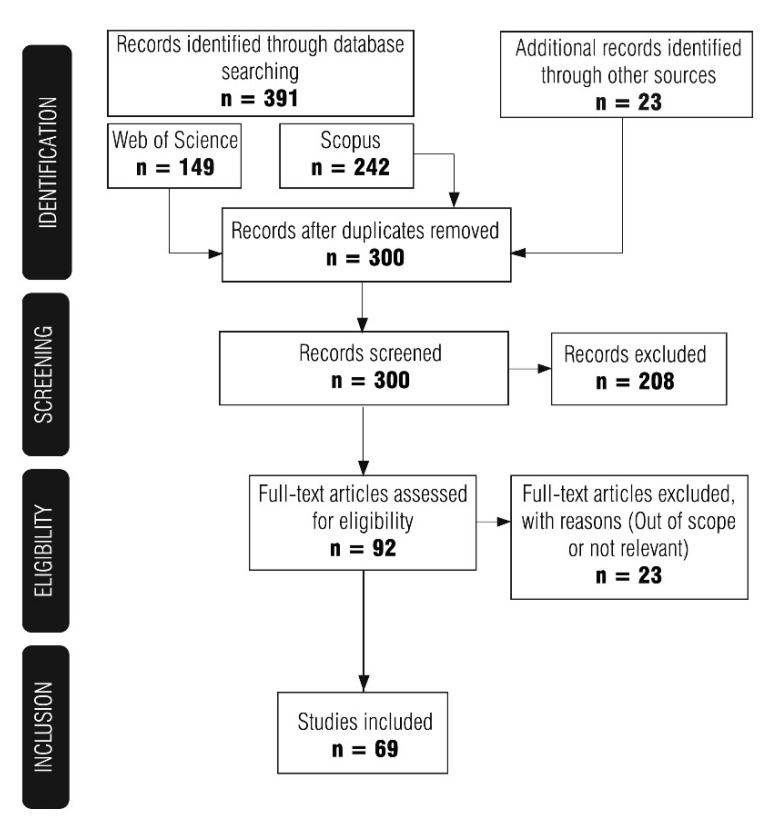
Steps in the review process for the selection of articles.

As a first step, a systematic literature search was conducted. Relevant studies were searched in the databases of Web of Science [29] and Scopus [30] over a period between 1 January 2000 and 30 October 2020. Initially, the authors began the search with the terms [(ground penetrating radar OR GPR) AND (corrosion) AND (concrete)]. The authors found that a number of studies for on-site assessment of concrete structures using GPR were excluded. They suggest that this is because some of the studies looked at the causes and consequences of corrosion (e.g., delamination), and it appears that the term corrosion was not appropriate in this case. For this reason, the term deterioration was included in the database search. The authors found that the terms [(ground penetrating radar OR GPR) AND (corrosion OR deterioration) AND (concrete)] expanded the number of studies so that a better overview of GPR application could be created. Duplicates were then removed, and the authors briefly reviewed titles and abstracts and excluded studies that did not meet the following criteria: (a) the study is published in English, (b) the full version of the study is available to the authors and (c) GPR was used to evaluate reinforcement corrosion and corrosion consequences (e.g., studies in which GPR was used only to determine cover thickness were excluded). Full-text articles were obtained, and further selection excluded studies that were not relevant or were beyond the scope. The final selection included 69 studies. Figure 1 shows the steps described.

## 3. Corrosion Monitoring Using Ground-Penetrating Radar

As mentioned earlier, the main causes of degradation are mainly the result of corrosion of reinforcement [31]. The corrosion of steel in concrete is a balanced electrochemical mechanism [32] between anodic and cathodic reactions that occur on the surface of the reinforcing steel. The anodic reaction, the oxidation of iron, occurs in an environment where the protective passive film of steel is not stable. The instability of the protective layer is related to the changes in the surrounding concrete and the main cause of these changes are processes such as chloride penetration or carbonation [32,33]. The time required for the breakdown of the passive film is called the initiation period in Tuutti’s corrosion model [34]. The further development of corrosion is called the propagation period and involves crack initiation, as a result of expansive stresses around the bar induced by rust formation. The progressive corrosion leads to spalling of the concrete and reduction of the cross-section of the reinforcement, which may compromise the load-bearing capacity of the structure [35]. Most corrosion assessment techniques are electrochemical-based [36,37]. In the field assessment of corrosion probability, the half-cell potential (HCP) and electrical resistivity (ER) are most used.

The description of the half-cell method and interpretation of the results are given in ASTM C876 [38] and RILEM recommendations [37]. The Wenner probe is commonly used to determine the electrical resistivity [39,40]. The resistivity values can be used to estimate the corrosion risk [41]. Although these methods have long been used successfully in the condition assessment of concrete structures, they have some drawbacks. The half-cell potential is a semi-destructive technique, so it requires a connection to the reinforcement. This is a limitation when a large area is to be inspected. Measuring electrical resistivity does not provide information about the reinforcement, only about the corrosive environment. Also, large areas require a lot of time for inspection. These issues can be overcome by using GPR.

**Figure 2:**
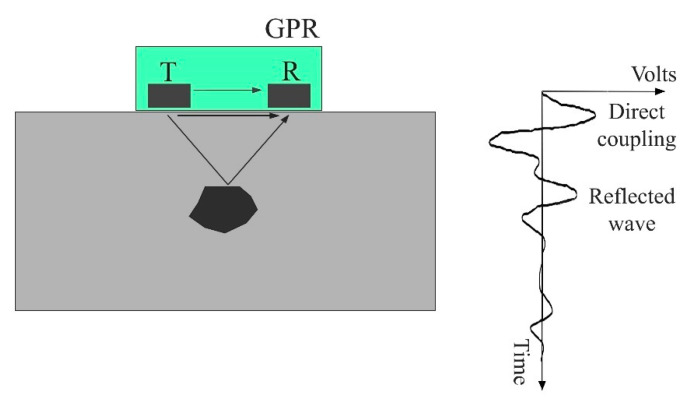
Recorded signals for ground-coupled antenna.

Ground-penetrating radar is a non-destructive technique that emits electromagnetic waves into the material, with the main objective of locating the buried objects underneath the surface [12]. Nowadays, its scope broadens to a wide range of materials, and among others is concrete [26]. The emitted electromagnetic wave propagates through the host material, as far as it encounters an interface between different materials, whereupon it is reflected back. The predominant types of GPR antennas used for civil engineering investigations are air-coupled and ground-coupled. The second type implies contact of the antenna with the ground and has a better penetration depth. The reflected wave is recorded with the receiving antenna and the recording is called an A-scan (Figure 2, right). When a wave is transmitted, the receiver first records a direct wave propagating through the air from the transmitter to the receiver. Then, a portion of the electromagnetic wave is reflected off the surface of the material. In a ground-coupled system, these two components superimpose to form the wave, called direct coupling, Figure 2. The rest of the wave energy passes through the material until it reaches the material with different dielectric properties. The electromagnetic wave is then reflected, and the receiver records it as a reflected wave. Therefore, the attributes of the A-scan that provide information about the target are the amplitude of the reflected wave and the travel time from the transmitter to the receiver. In addition, the most common representation of the results obtained with GPR is a B-scan, the two-dimensional slice that represents the area under investigation along the line.

The strength of the reflected wave depends on the properties of the host material. The properties that determine the behavior of electromagnetic waves in the material are its dielectric properties—dielectric permittivity (ε) and electrical conductivity (σ) [42]. Signal losses are mainly due to electrical conduction and dielectric relaxation [43]. Electrical conduction arises from the motion of free charges, while dielectric relaxation arises from the rotation of polar molecules. At the microscopic scale, friction occurs between particles due to these motions, resulting in energy dissipation. In summary, the propagation behavior of electromagnetic waves strongly depends on the composition of the pore solution. Changes in dielectric properties can be expected in the presence of moisture and/or chlorides in the concrete. The presence of water molecules and chlorides in pores results in an overall loss of energy and signal [43,44,45]. However, this attenuation is primarily caused by the presence of chlorides in the pore solution [46] as a result of the increased electrical conductivity of concrete. Changes in the concrete microstructure caused by carbonation can also affect the GPR response. The most noticeable ones are due to a reduction in porosity and ion exchange in the pore solution. It has been reported that carbonation causes a decrease in the dielectric permittivity, resulting in reduced attenuation [47].

### 3.1. Laboratory Simulated Corrosion Inspection

Corrosion assessment using ground-penetrating radar is still a novel approach, therefore only a limited number of studies have been conducted under laboratory conditions (Table 1). There are many challenges to ensure a suitable experimental setup for such a study, starting with the criteria for corrosion initiation, corrosion monitoring and corrosion probability assessment. In order to simulate natural corrosion under laboratory conditions, various techniques are often used to accelerate the process, such as impressed current technique, artificial climatic environment, accelerated migration tests, etc. [48]. The most commonly used method for corrosion acceleration is the impressed current technique, which is based on exposing the embedded reinforcement to the electric current provided by an external power supply. The current density and exposure time are controlled to achieve different degrees of corrosion [49,50]. Besides, corrosion can be enhanced by creating favorable conditions such as high temperature, high humidity and cycles of wetting–drying [51]. Even if these methods tend to simulate corrosion well, it is inevitable that the artificial conditions for corrosion to occur will differ from natural conditions. Recognition of these limitations is important to ensure adequate correlation between accelerated corrosion investigations and on-site corrosion assessments using GPR. Therefore, Table 1 summarizes the corrosion probability studies conducted to date that consider both corrosion acceleration methods and GPR signal attributes analysis. Two experimental setups were found: (a) GPR attributes acquired before and after the corrosion process, and (b) GPR attributes monitored during the corrosion acceleration process. The second setup is more significant as it ensures information about the different stages of corrosion, starting from the depassivation of the steel to the appearance of cracks.

One of the first studies to have acknowledged the GPR potential for corrosion detection was published in 2003 by Hubbard et al. [16]. The rebar was subjected to an accelerated corrosion process for 10 days. The results showed that corrosion causes a reduction in amplitude, which they attributed to the scattering and attenuation of waves due to the roughness at the corroded interface between concrete and rebar. This was also confirmed in Reference [52], where the amplitude of the signal was also reduced in specimens where the previously corroded bar was cast in concrete. To extend these observations, additional specimens were subjected to an accelerated corrosion process in an environmental chamber at different corrosion levels. It was found that corrosion causes a decrease in amplitude for each corrosion level. This is explained by the signal scattering in the concrete cover zone caused by the presence of cracks, corrosion products and the roughness of the corroded bar. In Reference [53], the influence of the diameter of the anode bar on the signal reflection was also observed. The increase in amplitude was associated with corrosion development but was also increased with the increase in diameter. In contrast, Reference [54] attributed lower amplitudes to the presence of corrosion products, but also indicated that the decrease could be influenced by the accumulation of chlorides in the concrete cover zone.

In Reference [55], concrete properties were simulated by oil emulsions with different dielectric permittivity, where corroded bars were immersed in the emulsions. However, in such an experimental setup, all results are based on the theories and therefore cannot faithfully represent real structures.

#### 3.1.1. Long-Term Corrosion Monitoring

Long-term monitoring of corrosion may ensure a better understanding of its effect on GPR signal attributes. Several studies [17,18,19,20,56,57,58] have been conducted to distinguish and correlate significant attributes of corrosion development and GPR signal. The conclusions from these studies are divided into: (1) initiation phase, (2) formation of cracks and (3) spalling of concrete cover.

##### Initiation Phase

The application of electrical current in the accelerated corrosion process induces the faster motion of chloride ions due to the electrical potential gradient, and this mechanism is called migration [32]. As this process thrives, a decrease in GPR amplitude is reported by Lai et al. [17]. According to the authors’ assertation, the accumulation of chloride ions around the anode absorbs the energy of the electromagnetic wave, which also causes the delay of the wave.

##### Formation of Cracks

After the initiation phase, the researchers had noticed a steady trend of change in the signal’s attributes until the wide crack is visible on the concrete surface. This phase is characterized by the formation of corrosion products that migrate into the surrounding concrete. The increased amplitude of the reflected wave was reported in Reference [57]. The rebars were subjected to external power supply until a longitudinal crack was visible on the concrete surface. The authors claimed that the migration of corrosion products into the shallower concrete cover zone enlarges the intersection points of the signal with different interfaces—concrete, microcracks, corrosion products—and leads to the increased amplitude. This was also confirmed in References [17,56,58]. In Reference [18], the experiment was set up to exclude the effects of moisture and chlorides on the GPR signal. The specimens were stored for two months to achieve stable moisture and chloride content before accelerated corrosion. Here, the increased amplitude of the GPR signal was then attributed to the effect of corrosion only. This was also outlined in Reference [20].

##### Spalling

In addition to the effect of corrosion development on the GPR amplitude, the effect of crack propagation and the occurrence of wide cracks on the amplitude of the GPR signal was also noted by Lai et al. [56]. A decrease in amplitude was observed after the occurrence of a wide longitudinal crack. This was explained by the scattering of signal energy caused by additional irregularities when a wide crack propagates through the concrete cover. The extension of the experimental setup was presented in Reference [20] to obtain a better representation of the crack influence on the signal amplitude.

#### 3.1.2. Conclusions from Laboratory Simulated Corrosion Inspection

**Figure 3:**
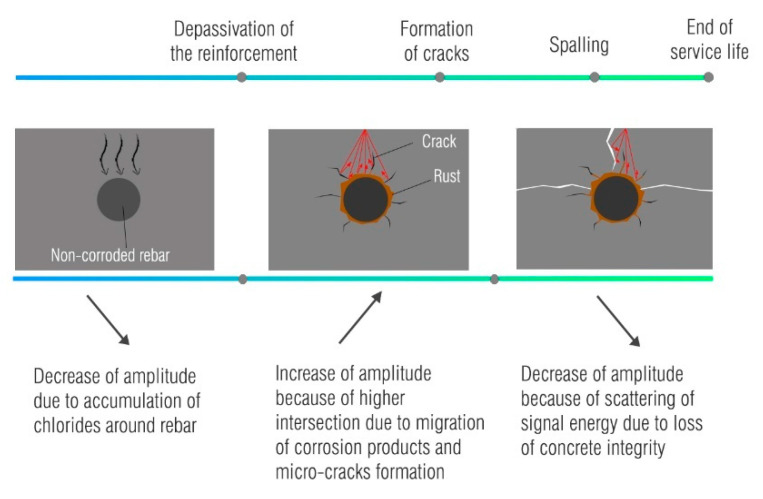
Changes in the GPR signal during corrosion process.

The corrosion process can be divided into the initiation and propagation phases, with each phase having specific effects on concrete microstructures. From the long-term corrosion monitoring experiments, the influence of corrosion promotion on GPR attributes was summarized, as in Figure 3.

The effects shown in Figure 3 are determined by the accelerated corrosion processes, in particular the formation of corrosion products on the surface of the reinforcing bar, their diffusion into the concrete cover and thus, crack propagation. These effects modify the amplitude in terms of different reflection coefficient and different dielectric properties of the concrete cover. The ability of corrosion products to migrate depends on the moisture content in the concrete cover since their movement is favored in the presence of moisture [59]. Similarly, the ability of their migration depends on the duration of the acceleration process. When accelerated corrosion with high current density is established in a short timeframe, it leads to increased crack width due to the sudden accumulation of corrosion products and increased pressure around the reinforcement [50,60]. Therefore, an appropriate current density should be selected to ensure the best possible simulation of natural conditions still within a reasonable timeframe [50,61].

The results of these studies also indicate that laboratory simulated corrosion studies mentioned above do not provide relevant results unless the level of corrosion is properly described. In these studies, results were recorded before and after corrosion acceleration and conflicting results were reported. Some authors reported higher amplitudes at the end of the experiments, while others reported lower amplitudes. Since these studies differ in terms of experiments setup, corrosion level and induced damage, it is possible that the observed changes in the GPR signal were recorded during different stages of the corrosion processes.

### 3.2. On-Site Corrosion Inspection

Most of the published research focuses on the application of GPR to the assessment of bridge decks, while other structures are represented to a lesser extent (tunnels, buildings, wharves, etc.). In terms of geographic location, most studies using GPR are conducted in the United States of America. The authors are sure that this is also a consequence of the existence of relevant standards [62].

The use of ground-penetrating radar data for condition assessment of concrete bridges dates back to the early 1980s [63]. The amplitude of the ground-penetrating radar signal during an inspection is affected by the presence of structural elements, variations in cover depth, moisture, chlorides [22,64,65] and other variables. Therefore, a simple approach to evaluating changes in the GPR signal is not practical. Coexisting influences with other phenomena, such as variations in moisture and chlorides, are unavoidable and prevent the development of methods for direct location of corroded rebar. Therefore, indirect methods are used in which the localization of corroded areas is correlated with the areas of high signal attenuation. Attenuation has been found to be strongly related to the increased conductivity around the rebar [22] caused by the accumulated chloride ions and corrosion products [25].

The inspection of concrete piers and wharves is very similar to the inspection of bridge decks where moisture and chlorides are the main causes of deterioration. The use of ground-penetrating radar has also been reported in the inspection of tunnels. It was noted that the complicated design and compound deterioration mechanisms of these structures made a simple corrosion assessment impossible. Instead, the data was used to assess the overall condition.

Quantification of attenuation can be determined by numerical analysis of the signal or by visual analysis of B-scans. These are discussed in more detail in the following sections.

#### 3.2.1. Numerical Analysis of GPR Attributes

The ASTM standard [62] for the evaluation of concrete bridge decks using ground-penetrating radar proposes two procedures for numerical analysis using GPR data. The first procedure is based on considering the reflection amplitude from the bridge deck bottom and the bridge deck surface. The second procedure considers the reflection amplitudes from the top reinforcement layer. In most cases, the reflection amplitudes from the top reinforcement are considered for corrosion evaluation. The amplitude is derived from the A-scan.

Numerical analysis is usually performed by normalizing the amplitude, which represents the deterioration rate, and is calculated as follows [66]:

(1) $$\mathrm{Normalized}\;\mathrm{amplitude}\left[\mathrm{dB}\right]=-20\log\frac{\mathrm{signal}\;\mathrm{amplitude}}{\mathrm{reference}\;\mathrm{signal}\;\mathrm{amplitude}\;}$$

The signal amplitudes are compared to the reference signal amplitude which is usually the amplitude with the lowest degree of attenuation and represents sound concrete [66]. The GSSI [67] suggests 32,767 for 16-bit data and 2,147,483,648 for 32-bit data as the reference signal amplitude. This approach may be inconvenient when a concrete structure is in an advanced stage of deterioration and high attenuation is primarily detected. The differences between amplitudes are then smaller and the deterioration could be underestimated [68,69]. On the other hand, if the structure is in a relatively good condition, the attenuation may be misinterpreted. In this case, the attenuation could come from a different source, namely the variation of the concrete cover thickness. In such cases, it is recommended to consider the whole amplitude and not only the attenuation zones [70]. Other approaches have also been used, with Dinh et al. [25] using the average direct coupling wave as the reference amplitude. According to Pashoutani et al. [71], the use of a constant value of a reference amplitude does not take into account the contribution of concrete surface quality to the signal amplitude, even to the normalized amplitude. Therefore, a normalization procedure was proposed in which each signal amplitude is normalized to its own direct coupling amplitude. In order to eliminate the influence of the cover depth variation on the signal amplitude, Barnes et al. [21] demonstrated an amplitude correction method. It was shown that subtracting the depth-dependent amplitude loss gives a better correlation of ground-penetrating radar amplitude maps with ground truth results than maps without correction. The method is based on determining the linear-dependent function of signal loss from the two-way travel time (TWTT) for the 90th percentile value of normalized amplitude. The 90th percentile value of the normalized amplitude is supposed to represent the sound concrete where the attenuation is mainly caused by the propagation of the signal through the dielectric material, i.e., the dielectric loss [25]. After correction of the amplitude, the attenuation should represent the signal loss due to chloride and moisture, i.e., the conductive loss. The method was improved after it was found that the conductive loss was also depth-dependent, so that an additional correction was necessary [25]. Two automated methods for depth correction have also been proposed [72]. In these studies, the correction was performed at the two-way travel time level. A more accurate correction could be performed if the linear function is determined using the real reinforcement depth instead of the two-way travel time [71]. This procedure requires the determination of the real velocities of the signal.

Obviously, it is of interest to establish a threshold for attenuation that is suitable for identifying the area of deterioration. However, a universally applicable threshold has not been established. It is usually based on the experience of the analyst and is related to a specific case [21]. There have been several attempts to relate the attenuation, mostly in comparison with thresholds of other methods. In References [73,74,75,76], ground-penetrating radar data were correlated with half-cell potential data, with the aim of determining the threshold value of attenuation. In Reference [75], the ROC (Receiver Operating Characteristic) curve was used, and in Reference [76], the author used statistical parameters to obtain the threshold value. In the second paper, the relationship between the percentage of corroded area, based on the results obtained with the half-cell potential at several bridges, and the product of the mean and skewness of the amplitude of the ground-penetrating radar, was established. The relationship can be used to predict the corroded area based on the analysis of the statistical parameters of the amplitudes. In some studies [77,78], the k-means clustering method was used to determine thresholds values.

**Figure 4:**
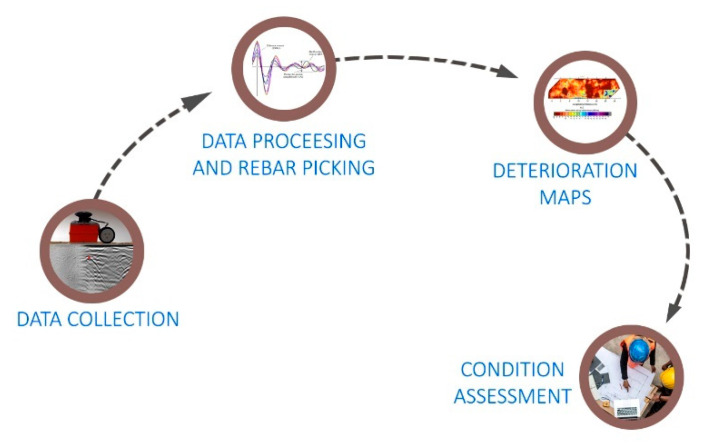
Numerical approach in condition assessment of concrete bridge decks.

Numerical analysis is generally used to obtain the deterioration map, which in most cases is the spatial distribution of normalized amplitudes. The main steps of the numerical approach in the condition assessment of concrete bridge decks are shown in Figure 4. The ability of the numerical approach to provide autonomous assessment of concrete structures using GPR is one of the reasons for its predominant use, while the algorithms for automatic reinforcement selection can be found in References [79,80].

The results obtained by periodical inspections can be collected in databases, so that the correlation of successive data allows continuous monitoring of the progress of deterioration. Dinh et al. [65] also proposed a method based on comparing the complete waveform (amplitudes and shapes of the electromagnetic wave) at a point with baseline data. The advantage of this method is that it excludes non-corrosion attenuation causes. However, baseline data is required for proper detection of deterioration, which is often not available. The method was improved in Reference [81] and the waveform was compared with the simulated waveform. The simulated waveform has the original direct wave, but it has no reflected wave, so it simulates full attenuation. The higher similarity with the simulated wave correlates with a higher degree of deterioration. Hong et al. [82] proposed a method to monitor the corrosion process by comparing different GPR data using the image registration technique.

Despite the deterioration maps, the statistical distribution of amplitudes had shown the relationship with the condition of the structure. In Reference [83], where several bridges with different environmental conditions were observed, it was found that after correcting the amplitude depth, the distribution of the amplitude of the sound deck was symmetrical with high kurtosis. In contrast, severely damaged concrete exhibited higher dispersion of amplitude distribution with lower value of kurtosis. This statistical dependence has been previously confirmed [74]. An automated crack tracking method based on the analysis of the processed amplitude of the ground-penetrating radar was presented in Reference [84]. The model considers the amplitude compared with the threshold value. The final result of the model is a three-dimensional (3D) visualization of the cracks, which provides the possibility to evaluate their geometry. However, the reliability of the model depends on the threshold value, which is difficult to determine accurately.

#### 3.2.2. Visual-Based or Combined Analysis of GPR Attributes

In addition to the numerical approach, a visual or combined visual and numerical approach has been supported by a number of authors [22,77,85]. The visual approach implies the visual analysis of B-scans. This method is highly dependent on the expertise of the analyst, especially in the case of severely damaged structures [86], so the final conclusion is prone to error. As noted by some authors [22], numerical analysis of amplitudes misinterprets most anomalies that alter the signal and are not causes of deterioration (surface anomalies, reinforcement spacing, reinforcement depth, structural variations). Due to of these drawbacks, a method is proposed in which an analyst reviews the ground-penetrating radar profiles (B-scans), considers the reflections of the reinforcement and concrete surfaces and marks the boundaries of deteriorated areas. The profiles are processed, and the final output is the corrosion map. The detailed procedure is described in Reference [87]. This method was improved to overcome the subjective opinion of analysts in visual-based interpretation [85]. A set of if/then rules was created to locate anomalies that alter the signal but do not indicate deterioration. Dinh et al. [77] used visual analysis of ground-penetrating radar profiles as a tool to determine the number of condition categories as input to the k-means clustering method. It is a combined method: after determining the number of condition categories, the amplitudes of the signal are grouped and thresholds between the groups are determined. The corrosion map obtained in this way was used for the deterioration modelling of concrete bridge decks presented in Reference [88]. Dawood et al. [89] presented an improved visual-based analysis of ground-penetrating radar data for the detection of air and water voids in tunnels. Moreover, an evaluation flowchart based on inspection of pier structure considering B-scans and GPR signal energy was proposed in Reference [90].

#### 3.2.3. Condition Assessment by Combination of Multiple NDT

Ground-penetrating radar has a number of advantages over other non-destructive techniques (NDT), and it is not surprising that it has shown much interest in replacing other techniques. It is completely non-destructive, and it is rational to give it precedence over other techniques that make surveying slow and less efficient. In the next sections, a brief overview is shown on current research results obtained by comparing GPR data with other test methods, such as electrical resistivity (ER), half-cell potential (HCP), chain drag (CD), hammer sounding (HS), infrared thermography (IRT), acoustic emission (AE) and impact-echo (IE). These studies are summarized in Table 2.

Electrical resistivity and half-cell potential are fundamental tools for determining the probability of corrosion in the condition assessment of concrete structures. A good correlation has been found in the analysis of electrical resistivity and GPR data [81,91,92,93]. However, such behavior is to be expected as both techniques are affected by the conductivity of the concrete [91].

The comparison between HCP and GPR data can be found in References [21,24,69,73,74,81,92,93]. All observations were obtained by superimposing the signal attenuation and potential maps. In most studies, a good correlation was found since the attenuation is indicative of a corrosive environment and coincides with the areas of extremely negative half-cell potentials [92]. However, when the degree of deterioration is low, the ground-penetrating radar could overestimate corroded areas [69].

Other techniques can also serve for condition assessment and correctly predict potential deterioration due to corrosion propagation. These techniques include chain drag (CD), hammer sounding (HS), infrared thermography (IRT), acoustic emission (AE) and impact-echo (IE). Compared to the chain-drag method, the ground-penetrating radar is effective while the deterioration level ranged between 10% and 50% [69]. However, the divergence between the result of the ground-penetrating radar and the acoustic scanning system was observed in Reference [94], where the authors investigated the suitability of these techniques for delamination detection. The area of high attenuation was larger than the delaminated area detected by the acoustic system because the ground-penetrating radar generally detects the deterioration earlier than the acoustic system. The GPR can detect deterioration before delamination occurs. Also, the comparative feasibility study on delamination detection using ground-penetrating radar (GPR) and infrared thermography (IRT) based on ROC (Receiver Operating Characteristic) analysis showed that IRT is more reliable than GPR in detecting delamination [95]. However, the contribution of IRT is limited to a shallow cover depth, while GPR can provide a deeper insight. Also, the usefulness of GPR in predicting repair quantities was presented in Reference [96], where the results of ground-penetrating radar matched the depth of removal measured by LiDAR (Light Detection and Ranging) method after hydro-demolition.

In a very detailed study, Omar et al. [109] presented the weaknesses and advantages of the most commonly used methods for condition assessment of concrete bridges. The conclusion was that none of the commonly used techniques are able to detect active corrosion, delamination and vertical cracking simultaneously, so that the most reliable condition assessment lies in a combination of multiple techniques. Such an approach ensures accurate condition assessment as deterioration can be detected from its onset to an advanced stage [23].

The simultaneously used non-destructive techniques usually consider methods such as ground-penetrating radar, electrical resistivity, half-cell potential, ultrasonic surface waves, impact-echo, etc. In References [14,100,110], an example of integration of different non-destructive testing methods in robotic systems, RABIT (Robotics Assisted Bridge Inspection Tool), was presented, which ensures real-time visualization of the concrete deck condition. Here, the evaluation is supported by a Jensen–Shannon probability method that focuses on the determination of the condition index [108]. Additional support in the interpretation of GPR data for delamination detection can be provided by infrared thermography (IRT) [99,103,104,107]. Solla et al. [106] demonstrated the technique to inspect a military base in an advanced stage of corrosion with visible signs of damage such as cracking and spalling. The results obtained with GPR were combined with the IRT technique. The corrosion assessment was based on the observation of GPR signal attenuation, changes in signal velocity and amplitude polarity. Overall, high signal attenuation was declared to indicate the presence of mineral salts and moisture, while reverse reflection polarity could be a sign of voids. The same parameters have been used in the assessment of wastewater plants [111], although the corrosion process is different in this case.

Deterioration modelling was part of the study in Reference [102], in which deterioration curves were developed based on the condition assessment of 10 bridges. Similar assessments were carried out by Alani et al. [101], where finite element models were constructed based on inputs from ground-penetrating radar and the deflection and vibration sensor system. In Reference [105], a data fusion model for bridge deck evaluation was developed based on the combination of the results from the ground-penetrating radar, half-cell potential method, electrical resistivity method and impact-echo method. In Reference [112], the ground-penetrating radar data combined with the capacitive technique and the impact-echo method were correlated with durability indicators for the overall assessment of the wharf.

#### 3.2.4. Conclusions from the On-Site Corrosion Inspection

**Figure 5:**
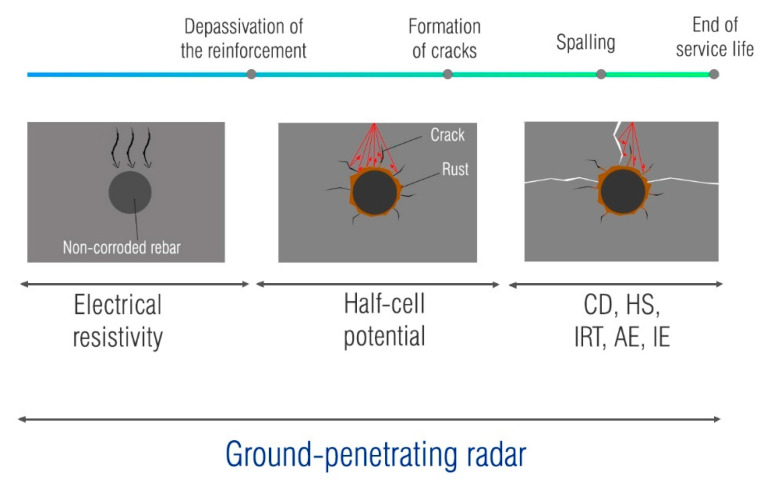
Multiple NDT used for the condition assessment.

The previous section has shown that corrosion assessment in on-site corrosion testing is mostly based on the assessment of the attenuated areas identified by signal amplitude analysis. Most of the studies are carried out on the bridge decks. In terms of comparison with other NDT, GPR has been compared with various techniques used for the service life condition assessment of the structures, Figure 5.

The high correlation between the electrical resistivity and the attenuation maps obtained with ground-penetrating radar is to be expected, as the signal depends on the material properties, so the conductive medium generated by moisture and chlorides changes its properties. In general, the GPR has shown good agreement with the HCP. However, there are certain situations where the GPR does not agree very well with the HCP. In cases where moisture and chlorides provide a favorable environment for corrosion, but their concentration is not sufficient to start corrosion, the GPR and HCP maps may differ. The applicability of ground-penetrating radar in detecting delamination is also uncertain [113]. In many cases, it does not detect delamination directly, and the assessment is based on the localization of deteriorated areas [114]. Moreover, the visual signs of delamination are not always visible on B-scans [87]. If the delamination is too thin to be detected by the antenna, it will not show any detectable change on the scan.

In summary, additional information, such as the age of the structure or the environmental conditions, may be helpful in analyzing GPR results. Moreover, this additional information can be obtained with other NDT, so a suitable combination of NDT can be a very powerful tool for the condition assessment of concrete structures.

## 4. Conclusions

This paper investigated the evaluation of corrosion probability in concrete using ground-penetrating radar. The study analyzed laboratory and on-site investigations and the results were related to the evolution of the corrosion process. Advantages and recommendations for future research are presented below.

GPR is a completely non-destructive method, which gives it an advantage over other techniques for corrosion assessment of reinforced concrete. Its ability to examine large areas in a short time, together with providing information on the depth and spacing of reinforcement, makes it a multifunctional NDT. The literature review identified certain challenges in the use of GPR for corrosion assessment, one of the main being the understanding of the influence of concrete conditions on GPR parameters. In fact, in most laboratory studies, moisture and chloride content were controlled after depassivation of the reinforcement. On-site in real conditions, variations of moisture and chloride content are inevitable, which makes the detection of corroded areas based only on the observation of amplitude potentially ambiguous. Since opposing data have been reported in the literature, further laboratory studies are needed to show the influence of the change in dielectric properties of the concrete cover on the GPR amplitude and the change in reflection coefficient due to the formation of corrosion products and their migration. Since an absolute comparison of studies is difficult due to the variance in experimental design and the degree of damage induced by the accelerated corrosion process, further studies should correlate the degree of damage with the change in GPR amplitude. Obtaining concluding results from the proposed research topics could enable the use of GPR as a stand-alone tool for detecting corroded areas, moving from its use for the detection of corrosive environment towards detection of corrosion itself.

In conclusion, as the knowledge of the effect of corrosion on the GPR signal increases, GPR will be a very valuable tool for condition assessment of reinforced concrete structures. This method will certainly be improved, leading to an upgrade of the construction management system and making the assessment more reliable with reduced maintenance costs.

## Figures and Tables

**Table 1:** Previous laboratory investigations on the influence of corrosion on the ground-penetrating radar (GPR) attributes.

Study	Year	Technique for Accelerated Corrosion Test	Method of Acquiring GPR Attributes	Current Density, i (µA/cm^2^)	Dimension of Specimens (m)	GPR (GHz) ^1^
Hubbard et al. [16]	2003	Impressed current technique	Before and after corrosion acceleration	-	1.25 × 1 × 0.25	1.2
Raju et al. [53]	2018			-	0.76 × 0.38 × 0.203	2.6
Zaki et al. [54]	2018			-	1 × 0.5 × 0.2	2
Lai et al. [56]	2010		Monitoring during corrosion acceleration	-	1.5 × 0.5 × 0.5	1.5 and 2.6
Zhan et al. [57]	2011			165,000	0.45 × 0.14 × 0.135	1
Lai et al. [58]	2011			340	-	1.5 and 2.6
Lai et al. [17]	2013			260 and 760	1.5 × 0.5 × 0.5	1.5 and 2.6
Hong et al. [18]	2014			424	1.5 × 1.5 × 0.3	2.6
Hong et al. [19]	2015			125	0.8 × 0.8 × 0.24	2.6
Wong et al. [20]	2019			650 ^2^	0.548 × 0.4 × 0.15	2
Hasan et al. [55]	2016	Corroded rebars immersed in emulsion	Before and after corrosion acceleration	-	Water oil emulsions	2.6
Sossa et al. [52]	2019	Corroded rebars cast in concrete		-	0.3 × 0.08 × 0.08	1.6
		Curing chamber		-	0.3 × 0.2 × 0.07	

^1.^ All of the antennas are ground-coupled. ^2.^ Level of current density was lowered in the latter stage of experiment.

**Table 2:** Review of studies that combined GPR with other techniques.

Study	Year	Other Techniques	GPR (GHz)		Main Findings
			Air-Coupled	Ground-Coupled	
Comparison with other NDT					
Barnes et al. [24]	2000	HCP, CD	1	-	Agreement on spatial distribution of deteriorated areas; 65.1% and 66.2% correctly predicted deteriorated areas compared to HCP and CD, respectively.
Scott et al. [97]	2003	IE, CD	2.4	1.5	GPR systems could not detect whole delaminated areas.
Barnes et al. [69]	2004	HCP, CD	1	-	GPR was effective in predicting damaged areas when the degree of deterioration is between 10% and 50%.
Rhazi et al. [73]	2007	HCP	-	1.5	The values for the degree of attenuation were proposed based on the correlation with HCP.
Barnes et al. [21]	2008	HCP, CD	-	1.5	The correlation between GPR and HCP and CD was improved after the depth correction.
Maser et al. [74]	2012	HCP, IE, HS	1 and 2	1.5 and 2.6	The agreement between GPR and HCP was 90.2%, and between GPR and IE was 79.3%.
Simi et al. [98]	2012	IE, CD	-	2	Moisture and corrosion maps produced with commercial software showed good spatial agreement with IE and CD.
Gucunski et al. [91]	2013	ER	-	1.5	The good agreement between GPR and ER; 95% of the locations where ER ≤ 40 kꭥcm agreed with the location where GPR amplitude was <15 dB.
Pailes et al. [93]	2015	ER, HCP, IE, CD, HS	-	1.5	The best spatial agreement compared to different NDT was between GPR and ER, and GPR and sounding techniques (CD and HS).
Dinh et al. [81]	2017	ER, HCP, IE	-	1.5	Correlation between GPR and other NDT was determined by a traditional numerical analysis and a method based on comparison with a simulated waveform; better agreement was found using ER and HCP than IE.
Sun et al. [94]	2018	AE, CD	-	1.5	GPR showed a larger deteriorated area than AE. GPR detected deteriorated areas near joints, while AE did not.
Sultan et al. [95]	2018	HS, IRT	-	1.6	Compared to the IRT, GPR was less accurate in detecting delamination.
Dinh et al. [92]	2019	ER, HCP	-	1.5	GPR maps produced by the method based on SAFT showed good correlation with HCP and ER. In one case, the correlation with ER was better than with HCP.
Combination with other NDT					
Maser [99]	2009	GPR, IRT	-	-	The combination of GPR and IRT was effective in condition assessment. The GPR assisted the IRT in detecting deeper delamination.
Gucunski et al. [23]	2010	GPR, ER, HCP, IE, USW	-	1.5	This combination of NDT can characterize different levels of deterioration. GPR brought effectiveness in the speed of inspection as the fastest technology from these five.
Gucunski et al. [100]	2013	GPR, ER, IE, USW	-	2	GPR deterioration maps were effectively implemented in a robotic system for bridge deck evaluation.
Alani et al. [101]	2014	GPR, deflection and vibration system	-	2	GPR results were combined with the deflection and vibration system to create a FEM model; GPR was used to locate rebar and detect cracks and potential moisture areas.
Kim et al. [102]	2016	GPR, ER, IE	-	2	GPR results were combined with ER and IE to calculate the condition index for estimation of service life.
Abu Dabous [103]	2017	GPR, IRT	-	1.6	Maps obtained with GPR and IRT were overlapped to form areas of possible delamination; the detected area was used to determine the condition rating.
Omar et al. [104]	2018	GPR, IRT	1	1.6	A method based on the integrated results obtained with GPR and IRT was proposed.
Ahmed et al. [105]	2018	GPR, ER, HCP, IE	-	-	Data fusion model from GPR, ER, HCP and IE maps was developed; fusion was on pixel and feature level.
Solla et al. [106]	2019	GPR, IRT	-	2.3	The paper proposes a procedure for anomaly detection based on joint observation of GPR signal and IRT temperature.
Kilic et al. [107]	2020	GPR, IRT, laser distance sensor, camera	-	2	The effectiveness of the integrated techniques was demonstrated on a bridge; GPR was used to detect water leakage, large cracks and corrosion.
Rashidi et al. [108]	2020	GPR, ER, HCP, IE, USW	-	1.5	The results from NDT were used to determine condition indices calculated using divergence from the ideal distribution using the Jensen–Shannon method.

## Data Availability

Not applicable.
